# Two Cases of Acute Diverticulitis Following Ocrelizumab Infusion in Patients With Multiple Sclerosis

**DOI:** 10.7759/cureus.52032

**Published:** 2024-01-10

**Authors:** Nicholas Vigilante, Cris S Constantinescu

**Affiliations:** 1 Department of Neurology, Cooper Medical School of Rowan University, Camden, USA; 2 Cooper Neurological Institute, Cooper University Hospital, Camden, USA

**Keywords:** drug-related side effects and adverse reactions, sigmoid diverticulitis, diverticulitis of the appendix, relapsing-remitting multiple sclerosis (rrms), ocrelizumab

## Abstract

Ocrelizumab is an anti-CD20 monoclonal antibody used to treat primary progressive and relapsing-remitting multiple sclerosis. Several prior case reports have demonstrated colitis in association with ocrelizumab infusion, and one case report has shown ocrelizumab-associated diverticulitis. We report on two cases in which ocrelizumab treatment of multiple sclerosis was complicated by acute diverticulitis. A 50-year-old woman and a 41-year-old man, both with relapsing-remitting multiple sclerosis, presented with acute abdominal pain. One patient had no known gastrointestinal history while the other had a history of laparoscopic sleeve gastrectomy. Both patients had received an ocrelizumab infusion one month prior to presentation. The woman underwent exploratory laparotomy, which revealed perforated sigmoid diverticulitis. The man was initially suspected of appendicitis and was treated with appendectomy, but a pathology review demonstrated diverticular disease in the appendix. In patients with multiple sclerosis on ocrelizumab, presentation with diverticulitis should include ocrelizumab-induced diverticulitis in the differential diagnosis.

## Introduction

Ocrelizumab is a humanized anti-CD20 monoclonal antibody approved to treat both relapsing-remitting and primary progressive multiple sclerosis (MS) via the selective depletion of B cells [1,2]. It is the only approved disease-modifying treatment for primary progressive MS [1]. While it is largely well-tolerated, ocrelizumab has been associated with several reports of colitis presenting weeks to months following an infusion. Some of these cases were managed medically while others required surgical resection [2-7]. There has also been one reported case of ocrelizumab-associated diverticulitis [8]. We report two additional cases of ocrelizumab-associated diverticulitis, both requiring surgical treatment.

## Case presentation

Case 1

**Figure 1:**
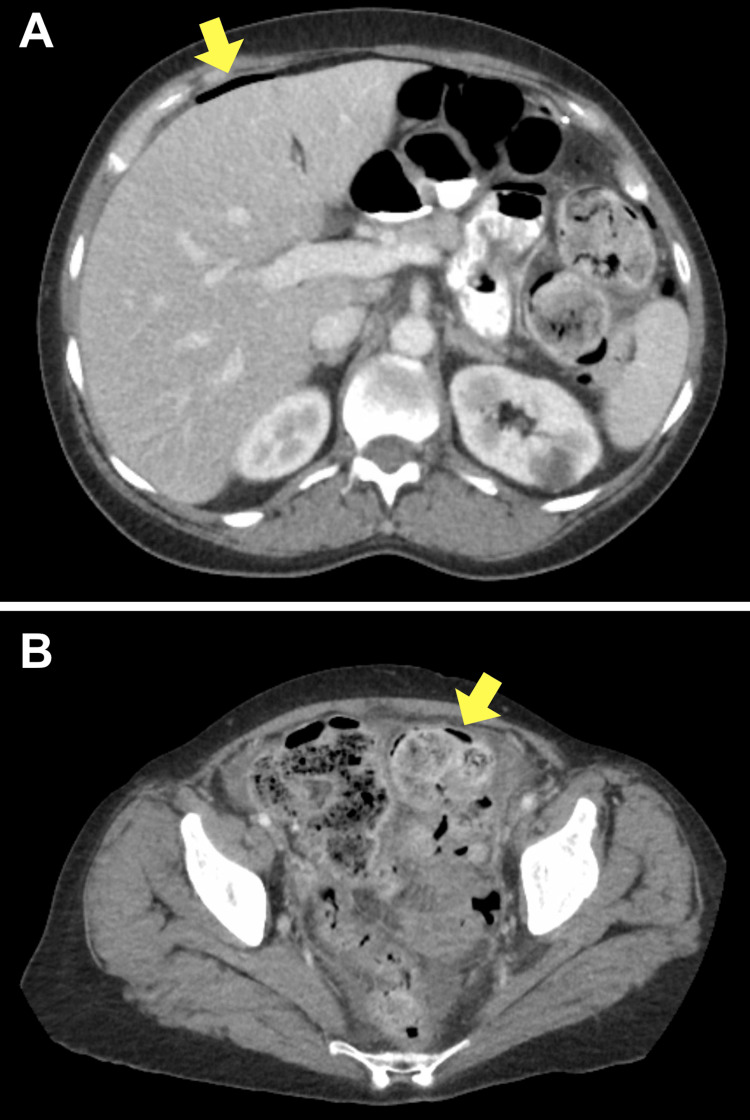
Abdominal computed tomography scan of a 50-year-old female with perforated diverticulitis (A) Axial image with abdominal free air anterior to the liver (arrow); (B) Axial image with wall thickening in the sigmoid colon and possible air in the sigmoid colon wall (arrow)

A 50-year-old woman with relapsing-remitting MS (RRMS), with a surgical history of laparoscopic vertical sleeve gastrectomy and no other known gastrointestinal history, presented to the emergency department with three days of progressive and diffuse abdominal pain. She was diagnosed with RRMS three months prior, having had symptoms for approximately two years, and her first infusion of ocrelizumab was one month prior. Physical examination revealed a distended abdomen with diffuse guarding and rebound tenderness. She was afebrile and hemodynamically stable. Initial laboratory studies demonstrated an elevated white blood cell count of 20.9 K/μL, and lactate, electrolytes, and creatinine were within normal limits. Urinalysis was negative for infection. Computed tomography (CT) of the abdomen and pelvis was significant for free air in the peritoneal cavity, free fluid in the pelvis, and thickening of the sigmoid colon wall (Figure 1). The patient underwent exploratory laparotomy which revealed appendicitis and perforation of the sigmoid colon; she subsequently underwent appendectomy and sigmoid colectomy with end colostomy (Hartmann procedure). Surgical pathology of the sigmoid colon showed pericolic abscess formation, diverticulosis, and diverticulitis with perforation. Pathology of the appendix showed appendicitis with fibrinopurulent exudate. The patient underwent Hartmann reversal six months later. At the neurology follow-up eight months after the index hospitalization, she denied any persistent gastrointestinal symptoms. Following a risk-benefit discussion with the patient, the decision was made to discontinue ocrelizumab and instead use ozanimod going forward.

Case 2

**Figure 2:**
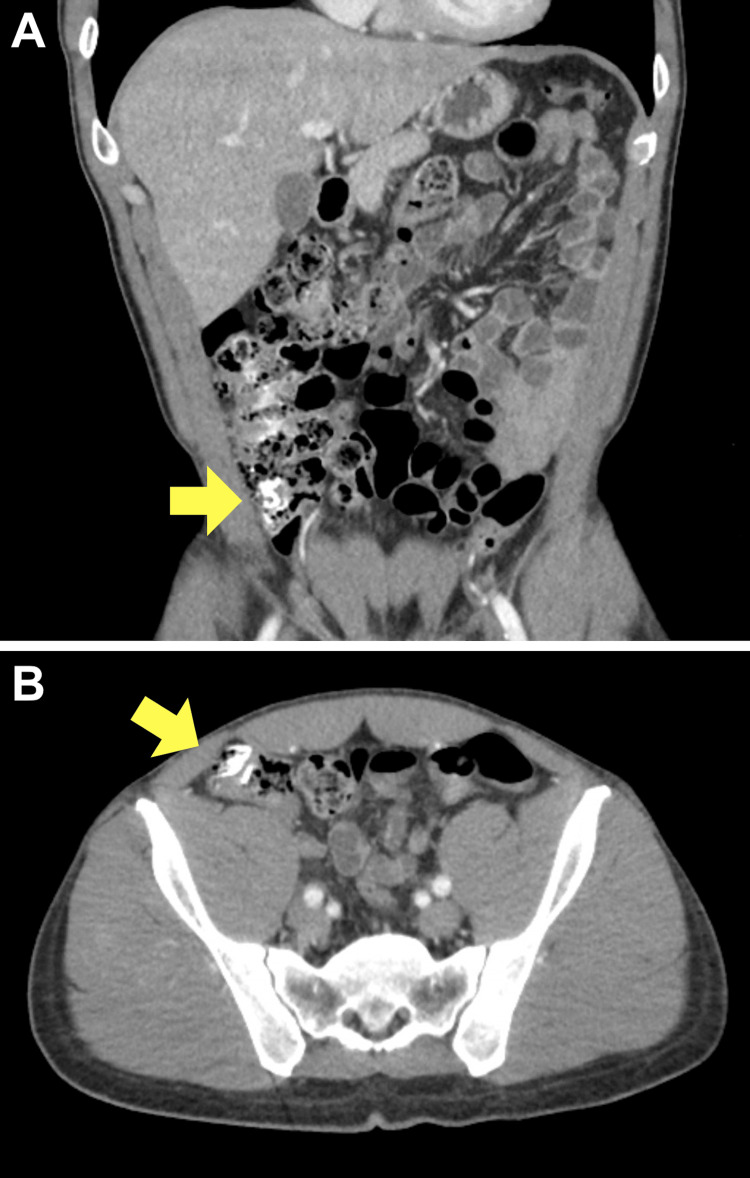
Abdominal computed tomography scan of a 41-year-old male with acute appendicitis Coronal (A) and axial (B) images of the dilated appendix with thickened, enhancing walls and periappendiceal fat infiltration (arrows)

A 41-year-old man with RRMS and no known gastrointestinal history presented to the emergency department with two days of epigastric abdominal pain and anorexia. He was diagnosed with RRMS 15 years prior and had been receiving ocrelizumab infusions for 18 months, with the most recent being approximately one month prior. His pain was not associated with nausea, vomiting, or diarrhea. The exam was significant for tenderness to palpation in the epigastrium, right upper quadrant, and right lower quadrant without rebound tenderness or guarding. He was afebrile and hemodynamically stable. Initial laboratory studies of complete blood count and basic metabolic panel were unremarkable. CT of the abdomen and pelvis was significant for a dilated appendix with a thickened, enhancing wall and no free fluid or air, consistent with acute uncomplicated appendicitis (Figure 2). The patient subsequently underwent laparoscopic appendectomy. Surgical pathology of the appendix revealed a sessile serrated polyp and diverticular disease with no evidence of neoplasm. At the neurology follow-up 10 months later, the patient denied any gastrointestinal symptoms. As the patient was both clinically and radiologically stable on ocrelizumab, the decision was made, along with the patient, to continue with ocrelizumab infusions, with regular gastrointestinal monitoring. At follow-up visits through two years after his appendectomy, the patient reported no persistent gastrointestinal symptoms.

## Discussion

We present two cases of diverticulitis in patients with RRMS on ocrelizumab. One patient required surgical management of perforated sigmoid diverticulitis while the other presented with diverticulitis in the appendix and required appendectomy. Following discussions with the patients, one patient remained on ocrelizumab following these events while the other switched to an alternative disease-modifying therapy.

Gastrointestinal symptoms are rare side effects of treatment with ocrelizumab, having been reported in 0.2% of patients in clinical trials and postmarketing data [9]. There are several prior case reports of patients with ocrelizumab-associated colitis, and one report of ocrelizumab-associated diverticulitis that presented with perforation [2-8]. In one prospective study in patients with RRMS, 0.5% of patients developed appendicitis while on ocrelizumab [10]. Prior reports of ocrelizumab-associated colitis have generally presented with days to weeks of bloody or non-bloody diarrhea. Some cases were resolved with corticosteroids while others were refractory to medical management and required colostomy. The reported case of ocrelizumab-associated diverticulitis also required surgical resection. These prior cases occurred in generally younger patients with no or limited gastrointestinal history, similar to the patients in this report. This pattern of presentation in younger patients may be due to the distribution of ages of patients starting ocrelizumab being younger than that of patients hospitalized with acute diverticulitis [11,12].

Both cases of diverticulitis that we present occurred approximately one month after the most recent ocrelizumab infusion. In one patient, this presentation followed their first infusion while the other patient had begun infusions 18 months prior. This timing is consistent with the findings in prior reports of ocrelizumab-associated colitis and diverticulitis, where patients presented over a range of two days to two months following their most recent infusion. Furthermore, of the cases reported in the literature, none reported having had more than three prior infusions [2-8]. However, while the time course following infusion and the presentation of these two patients have precedents in the literature, a chance association cannot be excluded.

One of the cases of diverticulitis presented herein was initially diagnosed as appendicitis and was later found to have diverticulitis within the appendix. While rare, appendiceal diverticulitis commonly presents by mimicking acute appendicitis and is generally diagnosed subsequently on review of pathology, much like in this patient [13,14].

The mechanism of ocrelizumab-induced colitis and diverticulitis is poorly understood. Depletion of B-cells in the intestinal mucosa may lead to local immune dysregulation through loss of intestinal immunoglobulin A (IgA), loss of local anti-inflammatory cytokines, such as interleukin 10, or dysregulation of intestinal T cells. This is supported by altered B-cell function being implicated in the pathogenesis of other forms of colitis such as inflammatory bowel disease [4,8,15]. Rituximab, another anti-CD20 monoclonal antibody, has also been associated with colitis and diverticulitis [8,16,17].

Of the two patients in this report, one continued treatment with ocrelizumab following his episode of diverticulitis while the other converted to an alternative disease-modifying treatment. In prior reported cases of ocrelizumab-associated colitis and diverticulitis, most patients discontinued ocrelizumab while one remained on ocrelizumab due to their clinical stability [2,3,6,8]. The cases presented in this report underline that either approach can be acceptable following a risk-benefit discussion with the patient. In the prior case of ocrelizumab-associated diverticulitis, the patient continued their ocrelizumab but with a modified dosing regimen. This patient underwent a segmental sigmoid resection and had no further episodes of diverticulitis [8]. This report underlines that patients may be able to continue their normal regimen without any additional or persistent symptoms. However, given the paucity of cases that have continued ocrelizumab therapy following colitis or diverticulitis and the subsequent difficulty in estimating the risk of recurrence, these patients should have ongoing gastrointestinal monitoring.

## Conclusions

Ocrelizumab is rarely associated with gastrointestinal adverse events such as colitis and diverticulitis. These cases may lead to significant morbidity due to the potential need for surgical resection. In patients with MS on ocrelizumab who develop diverticulitis soon after an infusion, ocrelizumab-induced disease should be considered in the differential diagnosis. This is especially the case for patients in atypical demographics for diverticulitis. In cases of appendicitis in patients with MS on ocrelizumab, pathology should be reviewed to determine whether diverticular disease is present in the appendix, which could signify ocrelizumab-induced disease.

## References

[1] Lamb YN (2022). Ocrelizumab: a review in multiple sclerosis. Drugs.

[2] Barnes A, Hofmann D, Hall LA, Klebe S, Mountifield R (2021). Ocrelizumab-induced inflammatory bowel disease-like illness characterized by esophagitis and colitis. Ann Gastroenterol.

[3] Sunjaya DB, Taborda C, Obeng R, Dhere T (2020). First case of refractory colitis caused by ocrelizumab. Inflamm Bowel Dis.

[4] Lee HH, Sritharan N, Bermingham D, Strey G (2020). Ocrelizumab-induced severe colitis. Case Rep Gastrointest Med.

[5] Akram A, Valasek M, Patel D (2020). P096 De novo colitis after ocrelizumab therapy. Gastroenterology.

[6] Malloy R, Fernandes R, Begun J, An YK (2022). Refractory fulminant colitis following ocrelizumab therapy requiring colectomy in a patient with multiple sclerosis. BMJ Case Rep.

[7] Tuqan W, Siddiqi F, Ray A (2020). Ocrelizumab-induced colitis: a case report. Am J Gastroenterol.

[8] Mehta DG, Wundes A, Strate LL, Romba MC (2021). Perforated diverticulitis associated with ocrelizumab infusion. Neuroimmunol Rep.

[9] Hauser SL, Kappos L, Montalban X (2018). Safety of ocrelizumab in multiple sclerosis: updated analysis in patients with relapsing and primary progressive multiple sclerosis. Mult Scler Relat Disord.

[10] Weinstock-Guttman B, Bermel R, Cutter G (2022). Ocrelizumab treatment for relapsing-remitting multiple sclerosis after a suboptimal response to previous disease-modifying therapy: a nonrandomized controlled trial. Mult Scler.

[11] Etzioni DA, Mack TM, Beart RW Jr, Kaiser AM (2009). Diverticulitis in the United States: 1998-2005: changing patterns of disease and treatment. Ann Surg.

[12] Hauser SL, Kappos L, Montalban X (2021). Safety of ocrelizumab in patients with relapsing and primary progressive multiple sclerosis. Neurology.

[13] Lesi OK, Probert S, Iqbal MR (2022). Diverticulitis and diverticulosis of the appendix: a case series. Cureus.

[14] Bujold-Pitre K, Mailloux O (2021). Diverticulitis of the appendix-case report and literature review. J Surg Case Rep.

[15] Uzzan M, Martin JC, Mesin L (2022). Ulcerative colitis is characterized by a plasmablast-skewed humoral response associated with disease activity. Nat Med.

[16] Rempenault C, Lukas C, Combe B (2022). Risk of diverticulitis and gastrointestinal perforation in rheumatoid arthritis treated with tocilizumab compared to rituximab or abatacept. Rheumatology (Oxford).

[17] Mallepally N, Abu-Sbeih H, Ahmed O, Chen E, Shafi MA, Neelapu SS, Wang Y (2019). Clinical features of rituximab-associated gastrointestinal toxicities. Am J Clin Oncol.

